# Diagnosis, Treatment and Risk Factors of *Strongyloides stercoralis* in Schoolchildren in Cambodia

**DOI:** 10.1371/journal.pntd.0002035

**Published:** 2013-02-07

**Authors:** Virak Khieu, Fabian Schär, Hanspeter Marti, Somphou Sayasone, Socheat Duong, Sinuon Muth, Peter Odermatt

**Affiliations:** 1 National Centre for Parasitology, Entomology and Malaria Control, Ministry of Health, Phnom Penh, Cambodia; 2 Department of Epidemiology and Public Health, Swiss Tropical and Public Health Institute, Basel, Switzerland; 3 University of Basel, Basel, Switzerland; 4 Medical Department and Diagnostics, Swiss Tropical and Public Health Institute, Basel, Switzerland; Yale Child Health Research Center, United States of America

## Abstract

**Background:**

Worldwide, an estimated 30 to 100 million people are infected with *Strongyloides stercoralis*, a soil-transmitted helminth. Information on the parasite is scarce in most settings. In semi-rural Cambodia, we determined infection rates and risk factors; compared two diagnostic methods (Koga agar plate [KAP] culture and Baermann technique) for detecting *S. stercoralis* infections, using a multiple stool examination approach; and assessed efficacy of ivermectin treatment.

**Methods/Principal Findings:**

We performed a cross-sectional study in 458 children from four primary schools in semi-rural villages in Kandal province, using three diagnostic procedures (Kato-Katz, KAP culture and Baermann technique) on three stool samples. Infected children were treated with ivermectin (100 µg/kg/day for two days) and re-examined three weeks after treatment. Hookworm, *S. stercoralis*, *Trichuris trichiura*, and small trematode eggs were most prevalent, with 24.4% of children being infected with *S. stercoralis*. The sensitivity of KAP culture and Baermann technique was 88.4% and 75.0%, respectively and their negative predictive values were 96.4% and 92.5%, respectively. The cumulative prevalence of *S. stercoralis* increased from 18.6% to 24.4%, after analyzing three stool samples, which was close to the modeled ‘true’ prevalence of 24.8%. Children who reported defecating in latrines were significantly less infected with *S. stercoralis* than those who did not use latrines (p<0.001). Itchy skin and diarrhea were significantly associated with *S. stercoralis* infection. The cure rate of ivermectin was 98.3%.

**Conclusions/Significance:**

*S. stercoralis* infection is highly prevalent among semi-rural Cambodian schoolchildren. The sensitivity of KAP culture is higher than that of the Baermann technique. In the absence of a “gold standard”, analysis of multiple stool samples by different diagnostic methods is required to achieve a satisfactory level of sensitivity. Almost three-quarters of the infections could have been avoided by proper sanitation. Ivermectin is highly efficacious against *S. stercoralis* but prohibitive costs render the drug inaccessible to most Cambodians.

## Author Summary

The difficulty of diagnosing *Strongyloides stercoralis* infections is the reason why up to date, accurate information on its geographic distribution in endemic regions and the total global burden is lacking. We conducted a cross-sectional study among 458 schoolchildren, with the purpose of comparing two methods for diagnosing *S. stercoralis* infection (Koga agar plate ‘KAP’ culture and Baermann technique) on three stool samples from each individual and to assess the efficacy of ivermectin three weeks after treatment. About one quarter of the schoolchildren examined were infected with *S. stercoralis*. The sensitivity of KAP culture and Baermann technique was 88.4% and 75.0%, respectively. The prevalence of *S. stercoralis* infection increased considerably (from 18.6% to 24.4%) when three stool samples were examined. Almost three-quarters of the infections could have been avoided by proper sanitation. Ivermectin was highly efficacious against *S. stercoralis* infection, with a cure rate of 98.3%. In the absence of a “gold standard”, it is necessary to examine multiple stool samples using different diagnostic techniques in order to reach a “true” prevalence.

## Introduction

The threadworm *Strongyloides stercoralis* affects about 30–100 million people worldwide [1], [2]. *S. stercoralis* is the only soil-transmitted helminth (STH) with the ability for auto-infection, and thus may lead to systemic infections with high parasite densities, particularly in immune-compromised hosts [3]–[5]. Disseminated strongyloidiasis may lead to severe complications with substantial mortality [4], [6].

Strongyloidiasis is endemic in areas where sanitary conditions are poor and where the climate is warm and humid [7], such as Central and South America, sub-Saharan Africa, and South and Southeast Asia [8]–[11]. However, little information is available on *S. stercoralis* prevalence in most of these settings. Today, most studies conducted in resource poor countries originate from Brazil and Thailand [12]–[15]. The sensitivity of widely used diagnostic procedures, such as direct fecal smear, Baermann technique and Koga agar plate (KAP) culture is not satisfactory when used on a single stool specimen [16]–[19].

The clinical manifestations of strongyloidiasis vary greatly in different situations, depending on infection intensities and the immune status of the individual. More than 50% of the infections may remain asymptomatic [20]–[22]. Gastrointestinal symptoms, including diarrhea and abdominal pain, are the most common symptoms [23], [24]. The most commonly described dermatologic aspects of chronic strongyloidiasis are itching and rash (urticaria) [25]. The recommended treatment for strongyloidiasis is ivermectin [26].

The detection of *S. stercoralis* larvae in the stool is proof of an infection [11]. Several diagnostic methods such as direct fecal smear, Baermann concentration, formalin-ethyl acetate concentration (FECT), Harada-Mori filter paper culture, or nutrient agar plate culture [27]–[30] have been used to identify larvae in stool samples. The exact sensitivity of these different diagnostic approaches is still debated [31]–[34].

In Cambodia, information on *S. stercoralis* infection is scarce. Earlier studies indicate prevalence rates of up to 20% in school children. However, they were assessed using a diagnostic approach with low sensitivity [16], [17], [35].

In semi-rural Cambodia, we determined infection rates and risk factors, compared two methods (KAP culture and Baermann technique) for diagnosing *S. stercoralis* infection using a multiple stool examination approach, and assessed efficacy of ivermectin treatment. We performed a cross-sectional study on *S. stercoralis* infection in four primary schools in semi-urban villages in Kandal province, examining three stool samples per child.

## Materials and Methods

### Ethical Considerations

The study was approved by the National Ethics Committee for Health Research (NECHR; number 033, dated 20 March 2009), Ministry of Health, Cambodia and by the Ethics Committees of the Cantons of Basel-Stadt and Baselland (EKBB; number 21/09, dated 29 January 2009), Switzerland. All relevant authorities (village chiefs, school teachers and headmasters) were informed about the purpose and procedures of the study. Written informed consent was obtained from the parent or the legal guardian of the child or appropriate literate substitutes, prior to study onset.

All diagnosed infections were treated according to Cambodian standard treatment guidelines [36]. All children infected with *S. stercoralis* were treated with ivermectin 100 µg/kg/day for two days [37].

### Study Setting and Population

The study was carried out in four semi-rural villages (Ang, Roka, Koh Khel and Damrey Chhlang villages), located in the Saang District (11.22°N and 105.01°E longitude), Kandal province, 45 kilometers south of Phnom Penh. Rice subsistence farming is the main economic activity in the villages. Pigs, poultry and cattle are the most common domestic animals. The villages were selected because hookworm infections were previously reported (used as a proxy for likely *S. stercoralis* transmission), and the villages were accessible by car to ensure rapid transfer of stool samples to the Parasitological Laboratory of the National Center for Parasitology, Entomology and Malaria Control (CNM) in Phnom Penh.

A school-based survey was conducted during the dry season from March to June 2009 among the schoolchildren of the four semi-rural villages mentioned above.

### Field Procedures

First, parents or legal guardian of the children were interviewed at home, using a pre-tested household questionnaire, to obtain the demographic data (age, sex, education level, profession), personal risk-perception (knowledge about helminth infections, health seeking behavior), living conditions (type of house, sanitation infrastructure, domestic animals) and personal hygiene.

Second, at school, a pre-tested child questionnaire was administered to the schoolchildren to obtain demographic data (age, sex, school grade), personal risk-perception (knowledge about helminth infection) and behavior data (wearing shoes, food consumption and personal hygiene) from the child. After the interview, each child received a pre-labeled plastic container (ID code, name, sex, age and date) for stool sample collection. Each morning, after collecting the filled container, another empty pre-labeled one was provided for the following day. This procedure was repeated until three stool samples were obtained per child or over a period of five days.

Within 90 minutes after collection, the stool samples arrived at the laboratory at ambient temperature. Upon arrival, experienced laboratory technicians from the Parasitological Laboratory of CNM immediately examined the specimens, as explained below.

### Laboratory Procedures

Stool samples were first subjected to a KAP culture, then a Kato-Katz thick smear examination and finally a Baermann technique was performed.

First, KAP culture [27] was used for identifying *S. stercoralis* and possibly hookworm larvae. For this purpose, agar plates were prepared once per week and stored at 4°C in humid conditions. A hazelnut-sized stool sample was placed in the middle of the plate and the closed Petri dish was incubated in a humid chamber for 48 hours at 28°C. Afterwards, the plates were rinsed with sodium acetate-acetic acid-formalin (SAF) solution. The eluent was centrifuged and the sediment microscopically examined for the presence of *S. stercoralis* and hookworm larvae. The two species were distinguished by the characteristic morphology of the larvae (i.e., size of buccal cavity, presence of genital primordium (L_1_), presence of forked tail-end (L_3_)).

Then, a single Kato-Katz thick smear [38] was prepared using the WHO standard template and examined under a light microscope for the presence of helminth eggs.

Finally, the Baermann technique [30] was performed. A walnut-sized stool sample was placed on gauze inserted into a glass funnel, and covered with water. The apparatus was exposed for two hours to artificial light directed from below. After centrifuging of the collected liquid, the sediment was examined under a microscope for presence of *S. stercoralis* larvae. If insufficient stool was submitted Baermann technique was dropped first.

For quality control, the technicians were specifically trained for three days on morphological criteria distinguishing hookworm and *S. stercoralis* larvae. During the whole study period, beside the permanent and rigorous supervision by a qualified microscopist from the Swiss Tropical and Public Health Institute (Swiss TPH), Basel, Switzerland, any unclear diagnosis was immediately discussed and solved with the qualified microscopist and study supervisor. Additionally, ten percent of the slides were re-examined by the same qualified technician from Swiss TPH. Slides yielding discrepant results were re-read by involved reader. A definitive infection(s) was found to be such by consensus.

### Follow-Up after Treatment of *S. stercoralis* Patients

*S. stercoralis* infected children were treated with ivermectin over two days (100 µg/kg/day). Stromectol 3 mg (commercial name of ivermectin), was manufactured on November 2008 by Merck Sharp & Dohme BV in the Netherlands. The drug was registered under no. 3523885; expiry date November 2011 (Manufactured batch: NK03350; Packed batch: NK18050). At 21–23 days after treatment, the infected children were asked to provide another three stool samples (over five days), which were then examined with the same procedure as at baseline (KAP culture, Kato-Katz and Baermann technique). Ivermectin treatment was provided under direct observation of a medical doctor. Adverse events occurring within three hours after treatment were recorded. Parents or legal guardian of the child were asked to report any adverse event occurring within a week after treatment to a medical doctor by telephone.

### Statistical Analyses

Questionnaire and laboratory data were double-entered in EpiData version 3.1 (EpiData Association; Odense, Denmark) and validated. Statistical analyses were performed with STATA version 10.1 (StataCorp.; College Station, TX, USA). Only schoolchildren with a complete record (three stool samples examined with all three methods and complete questionnaire information) were retained for analyses.

Prevalence, sensitivity (i.e., proportion of true positives identified as positive) and negative predictive values (i.e., proportion of un-infected children among negative results) of the different *S. stercoralis* diagnostic methods employed were assessed. A mathematical model developed by Marti and Koella [39] was used to estimate species-specific ‘true’ prevalence rates. This model employs the number of positive test results among stool samples submitted by the same person, to estimate the sensitivity of the diagnostic method and to calculate the number of stool samples needed for the test to be below a given percentage of false negative results. The procedure has been employed before to estimate the ‘true’ infection rates of soil-transmitted helminths, including *S. stercoralis* [33], [34], [40], [41]. All *S. stercoralis* positive children, regardless of the number of stool samples provided, were followed up after treatment.

Univariate logistic regression was used to associate infection status with demographic variables, hygienic status, and knowledge of the child's guardian and the recent medical history of the child. Population attributable fraction was calculated for significantly associated risks. *P-values* under or equal to 0.05 were considered as significant.

## Results

### Study Sample and Compliance

**Figure 1:**
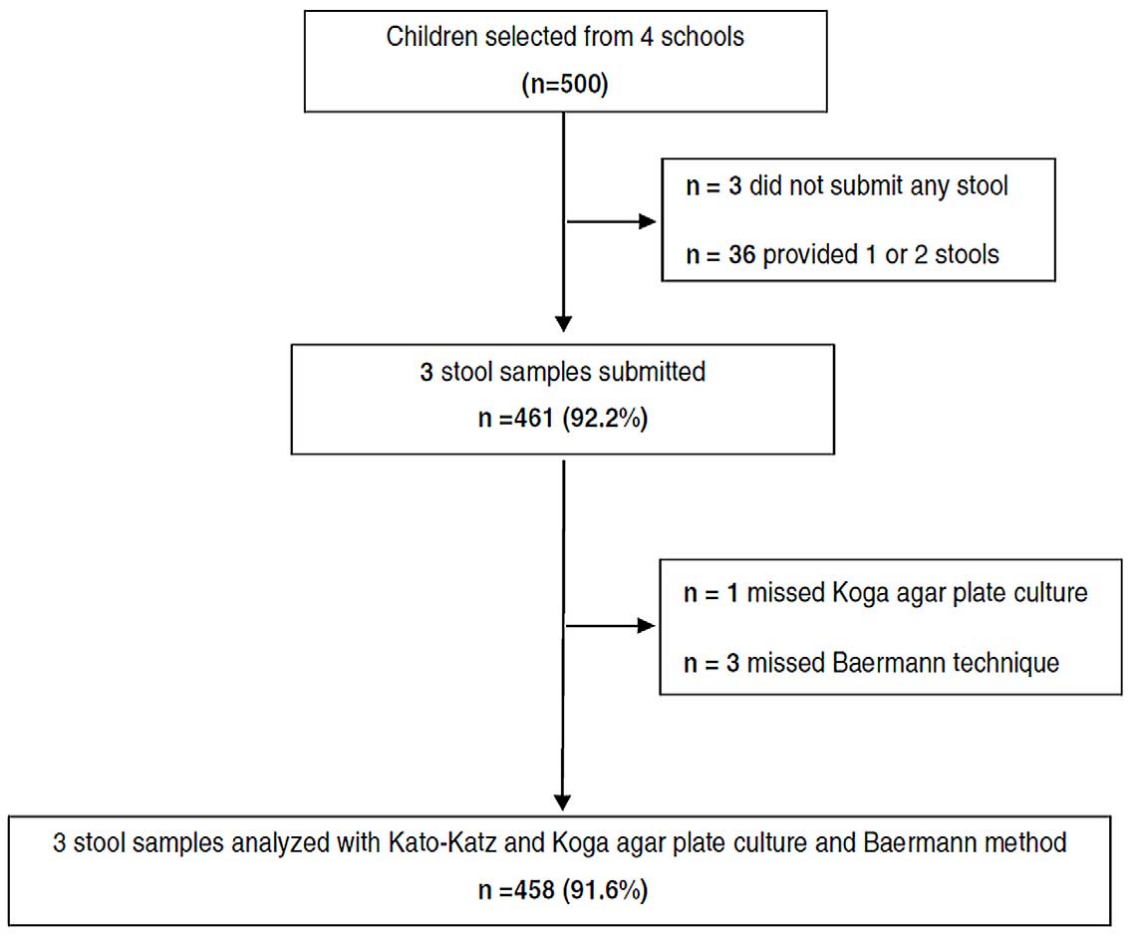
Flow chart detailing the study enrollment and compliance for stool examination, 2009.

In total, 500 children from four primary schools were enrolled (Figure 1), of which 461 (92.2%) submitted three stool samples over five days. The analysis focused on 458 (91.6%) schoolchildren with complete data records, i.e. three stool samples examined with all diagnostic tests. The participants were between 6 and 19 years old (median age 11 years); 227 (49.6%) were girls. There was no age difference between genders (p = 0.06).

### Parasitological Findings

**Figure 2:**
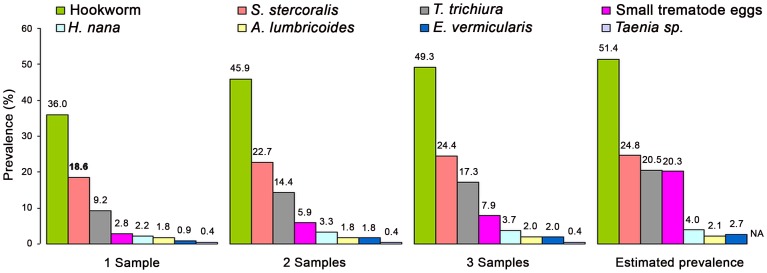
Observed cumulative and estimated prevalence of intestinal helminth infections among 458 schoolchildren in Cambodia, 2009. Legend: NA: Not Applicable; Koga agar plate and Baermann for *S. stercoralis*; Kato-Katz, Koga agar plate and Baermann for Hookworms; Kato-Katz for other infections.

The observed and estimated prevalence of eight different intestinal parasite species found from different stool samples and diagnostic methods are detailed in Figure 2. In total, half of the schoolchildren (49.3%) were infected with hookworm, and one quarter of them (24.4%) were diagnosed with *S. stercoralis* infection. *T. trichiura* was found in 17.3%, while 7.9% harbored small trematode eggs. Both, *A. lumbricoides* and *E. vermicularis*, were observed in 2.0% of participants, whereas *H. nana* and *Taenia* sp. were found in 3.7% and 0.4% of children, respectively.

### Performance of the Diagnostic Methods, Sampling Efforts and Prevalence Prediction

In total, 112 *S. stercoralis* infected schoolchildren (24.5%) were diagnosed by either KAP culture and/or Baermann technique out of 458 study participants who submitted three stool samples (Table 1). KAP culture and Baermann technique detected 99 and 84 *S. stercoralis* infections, respectively. The combination of the two tests was considered to be the “diagnostic gold standard”. The prevalence of *S. stercoralis* as estimated by KAP culture or Baermann technique alone was 21.6% and 18.3%, respectively. The sensitivity of KAP culture was 88.4%, whereas the sensitivity of Baermann technique was 75.0%. The negative predictive value of KAP culture and Baermann technique was 96.4% and 92.5%, respectively.

**Table 1:** Koga agar plate culture and Baermann method for the diagnosis of *S. stercoralis* in schoolchildren, 2009.

		Combined Methods (KAP culture and Baermann)		
		Positive	Negative	Total
**KAP culture**	**Positive**	99	0	99
	**Negative**	13	346	359
	**Total**	112	346	458
**Baermann method**	**Positive**	84	0	84
	**Negative**	28	346	374
	**Total**	112	346	458

The effect of the sampling effort for multiple stool samples examination on infection prevalence and on the sensitivity of the different diagnostic methods are presented in Table 2 for *S. stercoralis* and in Table 3 for hookworms. The number of *S. stercoralis* and hookworm infections detected by either method increased considerably by analyzing three stool samples. For *S. stercoralis* the prevalence rose from 15.9% to 21.6% and from 12.0% to 18.3%, as detected by KAP culture and Baermann method, respectively. The combined results of both methods showed an increase from 18.6% to 24.4% when three stool samples were examined instead of one.

**Table 2:** *S. stercoralis* larvae in Koga agar plate culture and Baermann method of three stool samples in schoolchildren, 2009.

	KAP culture		Baermann method		Combined 2 methods*	
	Number	%	Number	%	Number	%
Cumulative result after examination of						
1 stool sample	73	15.9	55	12	85	18.6
2 stool samples	90	19.6	78	15	104	22.7
3 stool samples	99	21.6	84	18.3	112	24.4
Estimated prevalence** (SD)		22.1 (3.9)		18.9 (3.7)		24.8 (4.1)
Sensitivity (3 samples)		97.7		97.3		98.6
Sensitivity of an individual test (SD)		71.3 (5.7)		69.9 (6.4)		76.0 (5.0)
Negative predictive value		99.3		99.4		99.6

* Koga agar plate culture and Baermann method.

** ‘Estimated’ prevalence according to a model developed by Marti and Koella [39].

SD: Standard Deviation.

**Table 3:** Hookworms in Kato-Katz, Koga agar plate culture and Baermann methods in three stool samples in schoolchildren, 2009.

	Kato-Katz Method		KAP culture		Baermann method		Combined 3 methods*	
	Number	%	Number	%	Number	%	Number	%
Cumulative result after examination of								
1 stool sample	115	25.1	79	17.3	55	12.0	165	36.0
2 stool samples	152	33.2	122	26.6	79	17.3	210	45.9
3 stool samples	169	36.9	142	31.0	99	21.6	226	49.3
Estimated prevalence** (SD)		39.0 (4.8)		36.7 (5.8)		30.2 (6.8)		51.4 (5.0)
Sensitivity (3 samples)		94.7		82.7		71.5		96.0
Sensitivity of an individual test (SD)		62.1 (4.9)		44.2 (6.2)		34.2 (7.7)		65.9 (4.1)
Negative predictive value		96.6		90.6		89.0		96.0

* Kato-Katz and Koga agar plate culture and Baermann method.

** ‘Estimated’ prevalence according to a model developed by Marti and Koella [39].

SD: Standard Deviation.

The ‘true’ *S. stercoralis* prevalence was estimated at 24.8% (SD = 4.1%) when the two methods were combined. The probability of correctly diagnosing *S. stercoralis* infected children by examining only a single stool sample was similar (70.0%) for both methods. The sensitivity and negative predictive value of all methods combined for three stool examinations was above 97% and 99%, respectively.

The predicted prevalence of hookworms was 51.4% (SD = 5.0%) when three stool samples were analyzed by a combination of all three methods (KAP culture, Kato-Katz and Baermann technique). The predicted prevalence for other intestinal parasites is shown in Figure 2.

### Efficacy of Ivermectin

Among all those children who submitted at least one stool sample (n = 497) during baseline examinations, 117 schoolchildren were diagnosed with a *S. stercoralis* infection. They were treated with ivermectin (100 µg/kg/day for two days). Three weeks after treatment, all children submitted two stool samples and 106 (90.6%) provided an additional third stool sample for examination. In two children, an infection with *S. stercoralis* was diagnosed (In one case, it was diagnosed in the first stool sample and in the other case, in the second stool sample. Both children were retreated with ivermectin.). No infection was detected in the third stool specimen. The cure rate of ivermectin was 98.3%. None of the children experienced an adverse event within three hours after treatment and none reported adverse events within a week after treatment.

The cure rates of treatments on the other intestinal helminth infections are presented in Table 4. Overall, 97 (82.9%) cases were co-infected with hookworm. Three weeks after mebendazole treatment (500 mg single dose), 65 (55.5%) participants were found hookworm positive in the follow-up examination. The cure rate of mebendazole was 33.0%.

**Table 4:** Cure rates of helminth infections among 117 *S. stercoralis* cases treated according to the Cambodian national guidelines, 2009.

Infection	Drug	Before treatment	After treatment	Cure rate
		n (%)	n (%)	
*Strongyloides stercoralis*	Ivermectin	117 (100.0)	2 (1.7)	98.3%
Hookworm	Mebendazole	97 (82.9)	65 (55.5)	33.0%
*Ascaris lumbricoides*	Mebendazole	2 (1.7)	0 (0.0)	100.0%
*Trichuris trichiura*	Mebendazole	38 (32.5)	1 (0.9)	97.4%
*Enterobius vermicularis*	Mebendazole	3 (2.6)	0 (0.0)	100.0%
*Taenia* spp.	Praziquantel	2 (1.7)	0 (0.0)	100.0%
*Hymenolepis nana*	Praziquantel	2 (1.7)	0 (0.0)	100.0%
Small trematode eggs	Praziquantel	18 (15.4)	1 (0.9)	94.4%

### Predictors of *Strongyloides stercoralis* Infection

Of 112 *S. stercoralis* cases, 108 (96.4%) were diagnosed in schoolchildren under 16 years, 42.0% were girls. As shown in Table 5, gender and age were not statistically different between infected and non-infected children. However, reported personal hygiene characteristics were significantly associated with *S. stercoralis* infection. The children who reported having shoes, and defecating in a toilet were half as likely to be infected with *S. stercoralis* than those who did not report shoes and latrine use (p<0.001). Itchy skin and diarrhea symptoms in the previous two weeks were reported more frequently among *S. stercoralis* cases.

**Table 5:** Risk factors for *Strongyloides stercoralis* infection in 458 schoolchildren from Cambodia, 2009.

	*S. stercoralis* Negative	*S. stercoralis* Positive	Relative Risk	Population Attributable Risk
	(N = 346)	(N = 112)		
	n (%)	n (%)	(95% CI)	(95% CI)
**Demographic characteristics of children**				
Gender (female)	180 (52.0)	47 (42.0)	1.3 (0.9–1.8)	
Age group				
6–10 years old	173 (50.0)	52 (46.4)	Reference	
11–15 years old	170 (49.1)	56 (50.0)	1.0 (0.7–1.4)	
>15 years old	3 (0.9)	4 (3.6)	2.4 (1.2–4.9)	
**Hygiene behavior of children**				
Defecates usually in toilet (yes)	160 (46.2)	26 (23.2)	2.2 (1.5–3.3)	0.4 (0.2–0.5)
Child washed hands after defecation, last time (yes)	237 (68.5)	47 (42.0)	2.2 (1.6–3.1)	0.3 (0.1–0.4)
Child washed hand before eating, last time (yes)	172 (49.7)	42 (37.5)	1.4 (1.0–2.0)	0.2 (0.01–0.3)
Child has shoes (yes)	306 (88.4)	77 (68.7)	2.3 (1.7–3.1)	0.1 (0.1–0.2)
Toilet at home (yes)	125 (36.1)	9 (8.0)	4.7 (2.4–9.0)	0.7 (0.5–0.8)
**Recent medical history of children (last 2 weeks)**				
Itchy skin (yes)	18 (5.2)	13 (11.6)	0.5 (0.3–0.8)	
Lost weight (yes)	23 (6.7)	7 (6.2)	1 (0.5–2.0)	
Nausea (yes)	26 (7.5)	7 (6.2)	1.1 (0.6–2.3)	
Vomiting (yes)	64 (18.5)	26 (23.2)	0.8 (0.5–1.2)	
Diarrhea (yes)	61 (17.6)	32 (28.6)	0.6 (0.4–0.9)	
Cold or cough (yes)	164 (47.4)	53 (47.3)	1 (0.7–1.4)	
Seen worm in stool (yes)	30 (8.7)	16 (14.3)	0.6 (0.4–1.0)	
Abdominal pain (yes)	226 (65.3)	67 (59.8)	1.2 (0.8–1.6)	
**Knowledge of child's guardian**				
Guardian reported child has been treated for Worm (yes)	216 (62.4)	45 (40.2)	1.9 (1.4–2.7)	0.3 (0.1–0.4)
Guardian knows about Worm/Worm Infection (yes)	258 (45.6)	25 (22.3)	2.3 (1.5–3.4)	0.4 (0.2–0.6)

Population attributable risk analysis showed that the number of strongyloidiasis cases would be reduced by 72% and 40% if all children had a toilet at home and used it for defecation, respectively. Bivariate analysis of population attributable risk showed that when the children in this population had toilet at home and defecated in it, strongyloidiasis cases could be reduced by 74%.

## Discussion

An in-depth parasitological investigation of *S. stercoralis* in Cambodia, including the performance of different diagnostic methods and the efficacy of treatment has not been carried out before. Our study confirms the validity of the KAP culture and Baermann method for detecting *S. stercoralis* larvae with high sensitivity and the high efficacy of oral ivermectin treatment (100 µg/kg/day for two days) in curing *S. stercoralis* infection. A cumulative prevalence of 24.4% was found among 458 schoolchildren in four semi-rural villages in Kandal province, south of Phnom Penh, by applying two methods on three stool samples collected over five days. This prevalence is substantially higher than those stated in three previous reports from Cambodia [16], [17], , and in reports from neighboring Laos and Thailand [14], [19]. This is most likely due to the fact that we used a much more rigorous diagnostic approach (number of stool samples, multiple diagnostic methods) than did the other studies, where it was common to examine a single stool sample with a single method. Nevertheless, a prevalence (20.2%) similar to the one found in our study was observed in 2006, among school-aged children living in villages bordering Tonlé Sap Lake, northern Cambodia [35] and using only the Baermann technique to analyze a single stool sample. This observation indicates that in villages close to Tonlé Sap Lake the true prevalence was actually considerably higher. It further underlines the reason why *S. stercoralis* is so often underdiagnosed.

The prevalence observed in our study area is particularly high when compared to other studies that used a similar diagnostic approach. In Zanzibar, Stefanie Knopp and colleagues diagnosed *S. stercoralis* in 10.8% of schoolchildren [34] and in China, Peter Steinmann and colleagues found a prevalence of 11.7% in the general population [33], [42].

In the scientific literature, no agreement seems to exist regarding the respective sensitivity of the KAP culture and Baermann method. [31], [33], [34], [43], [44]. In our study, the sensitivity of the KAP culture was higher than that of the Baermann method, which was also reported in the studies of de Kaminsky [31] and Glinz [44]. In contrast, in recent studies conducted in China [33], Zanzibar [34] and Uganda [43], the Baermann method detected up to more than an additional 20% of *S. stercoralis* larvae in terms of the observed cumulative prevalence. KAP culture requires expertise in distinguishing hookworm from *S. stercoralis* larvae, and it is not always easy to perform it in rural settings of developing countries. The Baermann method is less time consuming, but it needs a considerably larger quantity of stool, which might lead to compliance problems if an additional sample is required due to insufficient volume. Hence, neither of the methods is sufficiently valid on its own and ideally, the two should be combined whenever possible. Today, 30–100 million people are estimated to be infected with *S. stercoralis* [1]. Given the low sensitivity of stool examination techniques most commonly used, this figure is likely to be an underestimation of the true burden of infection.

Since a true “gold standard” is not available, the results of analyzing one stool sample with a single test may not be sufficient to reach an acceptable estimate of the “true” prevalence of *S. stercoralis*. To overcome this problem, Siddiqui and Beck [11] proposed to analyze a single stool sample by multiple diagnostic methods simultaneously. In our study, multiple stool samples were examined using multiple diagnostic methods, which considerably increased the observed prevalence of *S. stercoralis*, consistent with observations in previous studies [33], [34], [42]. Using this approach, the final prevalence observed was close to the “true prevalence” as estimated in a mathematical model [39]. Collecting a large quantity of multiple stool samples on consecutive days from children, who are at high risk of *S. stercoralis*, is always a challenge. The pointed diagnostic methods including molecular (PCR in stool sample) [45] and serological (copro antigen) methods [46], which need a small amount of feces specimen, might be alternative option for prevalence studies of *S. stercoralis*. These techniques, however, require further validation and might need further development before they can be recommended for wider use. However, their cost and sophistication might hamper their introduction in resource poor countries where *S. stercoralis* is most prevalent.

Ivermectin, the drug of choice for treatment of strongyloidiasis, was highly efficacious and shows few side-effects at a single dose of 200 µg/kg or 100 µg/kg/day for two days. Our results confirm observations of previous studies [14], [15], [37], [47]–[51]. However, to demonstrate full eradication of a *S. stercoralis* infection is difficult as it could have dropped temporarily below the detection level and increase thereafter. Therefore, short follow-up periods might overestimate the complete cure. However, in populations exposed to on-going *S. stercoralis* transmission a longer follow-up period bears the risk of a re-infection. Therefore, ideal efficacy assessments for drugs against *S. stercoralis* should be conducted in non-exposed populations.

Ivermectin is not included in the list of essential drugs of the Ministry of Health of Cambodia. Although there are at least two big pharmacies in Phnom Penh where the drug is sold, the extremely high price (USD 10 per tablet) excludes its wide-scale use.

One third of cases were clear of hookworm after three weeks of single dose mebendazole treatment. Our observed low cure rate of single dose mebendazole is coinciding with the recent control trial study among school-aged children in Lao PDR [52]. Our study did not determine the efficacy of ivermectin against other STH infections. Nevertheless, ivermectin has shown low efficacy against hookworm and *T. trichiura* infections, except *A. lumbricoides* [50].

It is not surprising that the age of participants was not associated with *S. stercoralis* infection. The literature explains that individuals can acquire the infection, usually at a young age, which persists until the time of diagnosis in adulthood, thus without further exposure to infected areas [9]. Nevertheless, we observed that the personal hygiene of children was a significant predictor for a *S. stercoralis* infection. *S. stercoralis* is mainly transmitted through skin penetration by the infective larvae from contaminated soil [53]. The transmission of strongyloidiasis could be interrupted by improving basic personal hygiene, such as defecating in a toilet and wearing shoes when in contact with soil. Our study found almost three-quarter of strongyloidiasis cases in the population could be prevented if personal hygiene (possession and use of latrine) was improved.

In conclusion, *S. stercoralis* is highly prevalent in rural Cambodian schoolchildren. Almost two-thirds of the infections could be avoided by proper sanitation. In the absence of a “gold standard”, the examination of multiple stool samples with different diagnostic methods is required in order to reach a reliable estimate of the prevalence. An adequate therapeutic regimen in the treatment of chronic uncomplicated strongyloidiasis is ivermectin at a dose of 100 µg/kg/day for two days. The availability and cost of ivermectin are critical issues in Cambodia.

## Supporting Information

Checklist S1 **STROBE checklist.**
(PDF)
